# The Neuroprotective Effect of Irisin in Ischemic Stroke

**DOI:** 10.3389/fnagi.2020.588958

**Published:** 2020-12-22

**Authors:** Yaqiang Liu, Chunhua Zhu, Jiahui Guo, Yonghong Chen, Chaoyue Meng

**Affiliations:** Department of Neurology, Second Hospital of Hebei Medical University, Shijiazhuang, China

**Keywords:** exercise, stroke, AMPK, mitochondrial dynamics, irisin

## Abstract

Irisin is a PGC-1α-dependent myokine that causes increased energy expenditure by driving the development of white adipose tissue into brown fat-like tissue. Exercise can improve irisin levels and lead to its release into the blood. In ischemic stroke, neurons are always sensitive to energy supply; after a series of pathophysiological processes, reactive oxygen species that are detrimental to cell survival via mitochondrial dysfunction are generated in large quantities. As a protein associated with exercise, irisin can alleviate brain injury in the pathogenesis of ischemic stroke. It is thought that irisin can upregulate the levels of brain-derived neurotrophic factor (BDNF), which protects nerve cells from injury during ischemic stroke. Furthermore, the release of irisin into the blood via exercise influences the mitochondrial dynamics crucial to maintaining the normal function of nerve cells. Consequently, we intended to summarize the known effects of irisin during ischemic stroke.

## Introduction

The incidence of stroke has increased rapidly over the past few decades, causing it to become one of the main causes of death and long-term disability worldwide (Huang et al., 2012; Katan and Luft, 2018). Especially in low- and middle-income countries, the incidence of stroke-related mortality is increasing, resulting in a high economic burden for both the patients and society (Karimi-Khouzani et al., 2017). Ischemic stroke accounts for ~80% of stroke cases (Lapchak and Zhang, 2017). Among all types of ischemic stroke, focal ischemic stroke is the most common. Focal ischemic stroke is caused by middle cerebral artery occlusion (MCAO) (permanent or transient), resulting in a lack of blood flow through the MCA. Defective blood supply can cause a shortage of glucose and oxygen in nerve cells, thus increasing reactive oxygen species (ROS) production and disrupting cell homeostasis. These complications lead to pathophysiological processes including excitotoxicity, oxidative stress, inflammation, apoptosis, and cell death (Khoshnam et al., 2017).

Mitochondria play a pivotal role in the pathophysiology of cerebral ischemic reperfusion. As highly dynamic organelles, mitochondria undergo morphological transformation through fission and fusion. In ischemic stroke, fission and fusion play critical roles in maintaining mitochondrial homeostasis when nerve cells lose blood supply. When mitochondria are damaged, fusion exerts a protective effect, allowing functional mitochondria to complement dysfunctional mitochondria through combining components between organelles. Fission is needed to create new mitochondria. However, excessive fission results in mitochondrial dysfunction (Li and Liu, 2018; Wang et al., 2020). In ischemic stroke, mitochondria are the main source of ROS. As a source of stress, excessive ROS damages the normal morphology of mitochondria, disrupting brain cells (Li and Liu, 2018). Therefore, maintaining mitochondrial integrity can serve as an alternative candidate for the development of neuroprotective tactics for treating cerebral ischemic injury.

Physical exercise can reportedly alleviate some of these pathophysiological processes. In addition, in the rehabilitation stage of stroke, exercise can also effectively improve sequelae symptoms (Li et al., 2017; Ryan et al., 2017). In 2013, studies reported that brain-derived neurotrophic factor (BDNF) is a possible mediator of the neurological benefits of exercise (Mang et al., 2013). BDNF is an abundant growth factor that is correlated with activity-induced neuroplasticity (Mang et al., 2013), and is upregulated by exercise in the animal brain (Berchtold et al., 2005; Rasmussen et al., 2009; Quirié et al., 2012). In a chronic stroke, treadmill high-intensity interval training elicited a significantly acute increase in BDNF (Boyne et al., 2019). Vascular endothelial growth factor (VEGF) is another neurotrophin that accumulates in human blood during exercise (Wahl et al., 2014). Peripheral increases in VEGF expression promotes perilesional angiogenesis and neurologic recovery in animal models of post-acute stroke (Zhang et al., 2000; Yang et al., 2010). Furthermore, several studies are substantiating the benefits of an exercise intervention on induced brain injury in animal stroke models (Ding et al., 2005; Matsuda et al., 2011; Sakakima et al., 2012; Otsuka et al., 2016). A recent study also demonstrated that the exercise-induced hormone irisin contributes to the neuroprotective effect of physical exercise against cerebral ischemia (Li et al., 2017).

**Figure 1:**
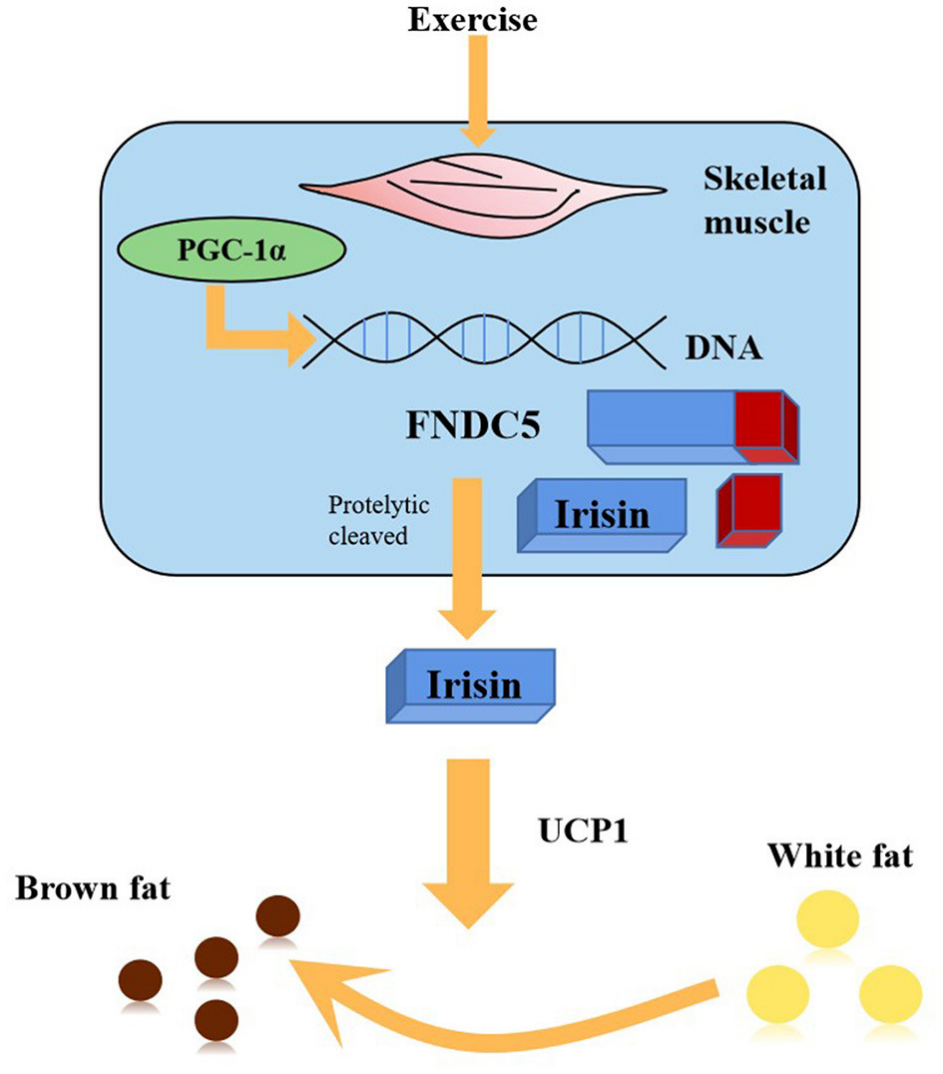
PGC1-α expression in muscle stimulates an increase in the expression of FNDC5, a membrane protein that is cleaved and secreted as the newly-identified hormone irisin. Irisin acts on white adipose cells in culture and *in vivo* to stimulate UCP1 expression and a broad process of brown fat-like development.

The previously unknown hormone irisin was discovered by Boström et al. (2012). Irisin is released into the blood through the enzymatic hydrolysis of PGC-1α after exercise, which could stimulate the transformation from mouse and human white fat cells into brown fat cells (Figure 1) (Boström et al., 2012). Since then, several studies have confirmed that irisin plays a protective role in the pathogenesis of many diseases, including neurodegenerative diseases, such as Alzheimer's disease, and cardiovascular diseases (Jin et al., 2018; Kim et al., 2018; Clark and Vissel, 2019; Conti et al., 2019; Young et al., 2019; Zhao et al., 2019). However, as research on the role of irisin in ischemic stroke is still limited, it is necessary to further elucidate its activity.

This review intends to summarize the structure and distribution of irisin in different tissues, the neuroprotective effect of irisin generation via exercise in ischemic stroke, and the correlation of exercise and irisin on mitochondrial dynamics. We, therefore, aim to provide a new research direction for exploring new treatment methods for ischemic stroke.

## Structure and Distribution of Irisin

Irisin was originally reported as a new hormone secreted from muscle cells upon exercise. It is encoded by *PGC-1*α, which is involved in many pathways related to energy metabolism (Boström et al., 2012). PGC-1α can stimulate skeletal muscle cells to express FNDC5 (a signal peptide with 29 amino acid residues), type III fibronectin assembly with 112 amino acid residues, and a C-terminal transmembrane domain with 65 amino acid residues. After the discovery of FNDC5 glycosylation by proteolytic enzymes, a new protein was identified. Irisin consists of 112 amino acid residues and a fibronectin III domain (Boström et al., 2012; Erickson, 2013). Previous research has revealed preliminary evidence that irisin is not only expressed in mammalian organs and tissues such as the heart, skeletal muscle, and brain (Aydin et al., 2014) but also in the thyroid, ovary, liver, lung, testis, esophagus, fat, and other tissues (Wrann et al., 2013). The distribution of irisin can also be detected in different brain regions and cell groups such as in Purkinje cells in the cerebellum (Varela-Rodríguez et al., 2016), astrocytes in the hippocampus (Piya et al., 2014), neurons in the brain (Wang et al., 2018), the hypothalamus (Dun et al., 2013), and even cerebrospinal fluid (CSF) (Aydin et al., 2013). Importantly, irisin has been demonstrated to have pivotal roles in attenuating inflammation, reducing oxidative stress, and alleviating apoptosis, as well as ameliorating mitochondrial dysfunction (Tu et al., 2020). Consequently, the discovery and distribution of irisin has provided a theoretical basis for exploring its effects in many diseases, especially in ischemic stroke.

## The Role of Irisin in Stroke

Skeletal muscle is a crucial organ in humans, accounting for ~40% of the human body weight. As the most energy-consuming organ, skeletal muscle accelerates the synthesis and secretion of muscle factors with active ingredients during exercise. These factors can act on other organs (such as the adipose tissue and the brain) in various ways by regulating sugar, lipid, and protein metabolism (Febbraio and Pedersen, 2005; Lee et al., 2015). Hence, the regulation of irisin/FNDC5 has obvious motor involvement.

The role of FNDC5/irisin in learning and memory is mediated by BDNF expression, which plays an important role in neural remodeling in conditions such as Alzheimer's disease (Wrann et al., 2013). A large number of studies have investigated the effect of exercise on irisin secretion. Exercise can upregulate BDNF levels in the hippocampus via PGC-1α activation and FNDC5 expression (Wrann et al., 2013; Xu, 2013; Yau et al., 2015). Experts have reached a consensus that BDNF may exert a neuroprotective role via irisin expression. For example, Islam et al. (2017) demonstrated that long-term exercise could increase BDNF expression in the brain through the PGC-1α-FNDC5 axis. It has also been shown that BDNF can enhance neuronal survival and migration (Raefsky and Mattson, 2017). Further research based on these studies demonstrated that BDNF expression was regulated by the application of irisin to a rodent stroke model during cerebral ischemia-reperfusion (Asadi et al., 2018). This study elucidated that BDNF is a crucial regulator of the beneficial effects conferred by irisin in ischemic stroke. As a protein that leads to irisin expression, the peripheral delivery of FNDC5 to the liver via adenoviral vectors could increase the level of BDNF and other neuroprotective genes in the hippocampus (Boström et al., 2012). This implies that irisin, or other factors induced by irisin, can cross the blood-brain barrier to affect gene expression in the brain. This discovery provides a theoretical basis for exploring the effects of irisin in ischemic stroke.

**Figure 2:**
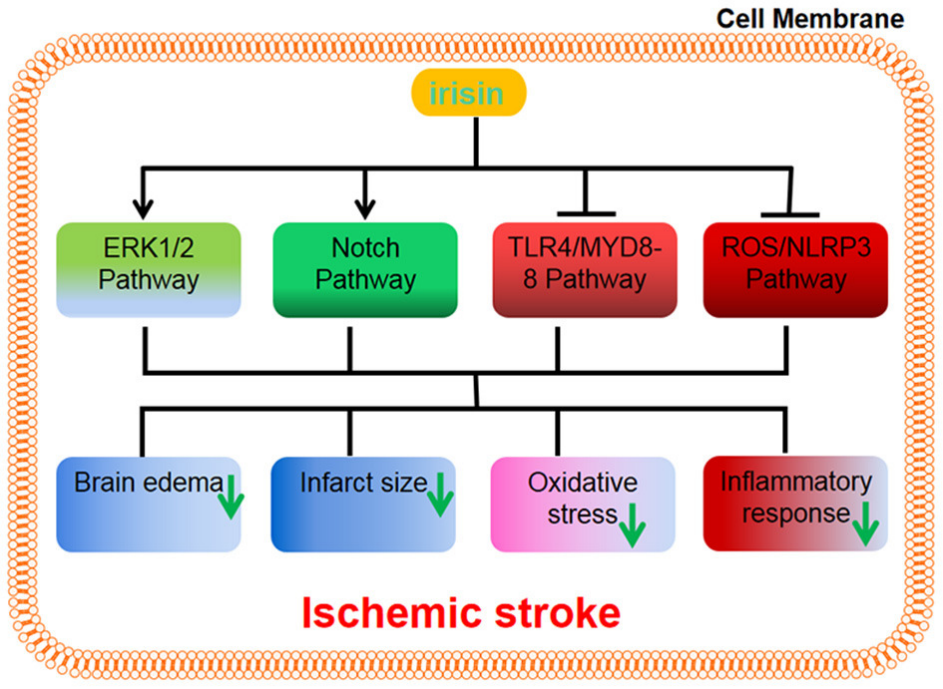
Irisin can exert a neuroprotective role by upregulating the ERK1/2 signaling pathway and Notch signaling pathway, and suppressing the TLR4/MYD88 pathway and ROS/NLRP3 pathway, respectively. The results reduce brain edema, infarct size, oxidative stress, and inflammatory response.

A recent study has reported that irisin protects the blood-brain barrier from ischemic injury by decreasing the expression of MMP-9 (Guo et al., 2019). Some research has demonstrated that exercise-induced irisin protects neurons from ischemia-reperfusion injury by reducing the volume of cerebral infarction, brain edema, and weight loss via Akt activation, which then leads to the activation of BDNF (Croll et al., 1999) and the ERK1/2 pathways (Li et al., 2017). Consistently, a recent study has indicated that brain edema and neurological function are alleviated by irisin during cerebral ischemia-reperfusion in mice and inflammation factors such as IL-1β and TNF-α are decreased and that apoptosis is reduced in the hippocampal neurons as a result of irisin treatment via activation of the Notch signaling pathway (Jin et al., 2019). Furthermore, irisin exerts a beneficial role *in vivo* (Peng et al., 2017) and *in vitro* (Yu et al., 2020) during ischemic stroke by suppressing the ROS/NLRP3 and TLR4/MYD88 signaling pathways, respectively. Together, these results suggest that irisin may exert a neuroprotective role during an ischemic stroke (Figure 2).

Notably, although Li et al. (2017) have found that in MCAO mice, irisin expression in the plasma is negatively associated with the concentration of pro-inflammatory cytokines IL-6 and IL-α, the transition of white adipose into brown adipose tissue may take some time, which is usually not available. Consequently, exogenous irisin treatment may be necessary during an ischemic stroke (Peng et al., 2017; Jin et al., 2019; Yu et al., 2020). Moreover, the patterns of exercise are very important for the generation of irisin. The forms of exercise include endurance and resistance exercise training; the former is an aerobic and cardiovascular form of exercise, while the latter focuses more on muscle strength and hypertrophy (Cornelissen and Smart, 2013; Ryan et al., 2017). At present, evidence suggests that irisin is involved in endurance exercise. This is to be expected as resistance training activates the PGC-1α isoform PGC-1α4, while endurance exercise regulates PGC-1α1, an upstream transcriptional regulator of FNDC5 (Ruas et al., 2012). Consistently, a study has also demonstrated that there is no difference in serum irisin levels between the control subjects and those who performed exercise, after the high-intensity interval and resistance training (He et al., 2018).

## Irisin and Mitochondrial Dynamics

As previously described, the discovery of irisin provides an alternative direction for studying the potential treatment methods for ischemic stroke. In 2017, Lidongjie et al. found that irisin synthesis reduces the infarct volume and the degree of brain edema and improves the neurobehavioral score in an oxygen-glucose deprivation model (Li et al., 2017). During physiological processes, the exercise-induced actin irisin can participate in energy metabolism by affecting mitochondrial function. Some studies have confirmed that when the regulation of energy metabolism is impaired, mitochondrial function is damaged, which further aggravates tissue damage in ischemia-reperfusion models (Chen et al., 2015; Flippo et al., 2018; Guo et al., 2018; Kim et al., 2018; Zabala et al., 2019; Zhang et al., 2019; Zhao et al., 2019). Exercise and irisin pretreatment exert a protective role by affecting the mitochondrial dynamics in tissues (Zhang et al., 2014; Chen et al., 2017; Bi et al., 2019). In addition, in the presence of ROS, exercise can maintain the normal morphology of mitochondria by activating AMPK (Trewin et al., 2018).

## Mitochondrial Dynamics and Stroke

Mitochondrial dynamics mainly consist of fission and fusion. Fission is mediated by the proteins Drp1, Fis1, and MFF. Drp1 is recruited from the cytosol to the outer membrane of mitochondria and interacts with its receptor proteins MFF and Fis1 to create the fission complex. Drp1 is then oligomerized into filaments that wrap around mitochondria, leading to mitochondrial constriction and sequential separation of the inner and outer membrane. Drp1 reportedly has a crucial role in ischemic stroke; brain edema, the infarct area, and other neuronal injuries are alleviated following Drp1 downregulation (Anzell et al., 2018; Kameoka et al., 2018).

Three different GTPases mediate fusion, including Opa1 and Mfn1/2. Mfn1/2 are anchored to the outer membrane of mitochondria, while inner membrane fusion is mediated by Opa1. A lack of mitofusins prevents fusion of both the outer and inner membrane of the mitochondria, while the loss of Opa1 only blocks fusion of the inner membrane. Mitochondrial fusion proteins are less studied in ischemic stroke. Mfn2 is reported to exert an anti-apoptotic effect, and its expression decreases in the presence of ROS. Opa1 can attenuate infarct volume in ischemic stroke, and its expression is increased after exercise (Anzell et al., 2018; Kameoka et al., 2018; Wei et al., 2019; Lai et al., 2020).

In ischemic stroke, cell survival and pathobiology are involved in mitochondrial dynamics. As mitochondrial dynamics processes, fission and fusion are crucial to mitochondrial function. Fusion is presumed to be beneficial to cell survival, but studies show that fission facilitates cell death (Li and Liu, 2018; Wang et al., 2020). Studies report that irisin can inhibit excessive Drp1-related mitochondrial fission and ROS, which exerts a protective role in ischemic disease (Bi et al., 2019; Tan et al., 2019). Furthermore, exercise can improve mitochondrial function in the brain by increasing the activity of the mitochondrial complex and Drp1 expression (Gusdon et al., 2017). Consequently, mitochondrial dynamics may have a key exercise-related role following ischemic stroke.

## AMPK and Mitochondrial Dynamics

It is reported that mitochondrial homeostasis is closely related to AMPK upregulation associated with altered cell energy metabolism (Siteneski et al., 2018). Animal studies show that irisin activates AMPK to inhibit liver cholesterol synthesis (Tang et al., 2016). Therefore, it is speculated that irisin may influence mitochondria by regulating AMPK expression.

AMPK is a heterotrimer including an α-subunit and two regulatory subunits, β and γ. The α-subunit is the main catalytic part of AMPK, containing a kinase domain and the key residue Thr172. When the ratio of ATP-AMP decreases, the AMPK complex is activated by phosphorylation on Thr172 in the α-subunit. The activated AMPK affects the mitochondrial dynamics by activating downstream substrates. When stresses, such as ischemia or hypoxia, are applied, the phosphorylated AMPK directly phosphorylates MFF. MFF then recruits Drp1 to the mitochondrial membrane, selectively causing fission of the damaged mitochondria and protecting normal mitochondrial function (Wang and Youle, 2016; Herzig and Shaw, 2018).

It has been reported that exercise is a potential activator of AMPK, demonstrating the possibility that AMPK can affect mitochondrial dynamics via exercise (Trewin et al., 2018). Moreover, irisin is necessary for mediating AMPK activity (Fan et al., 2020). Notably, AMPK reactivation can attenuate hyperglycemia-mediated mitochondrial injury. In heart ischemia, irisin can improve the expression of mitochondrial fusion proteins Opa1 and Mfn1 by activating the AMPK signaling pathway; blocking the AMPK pathway inhibits the regulatory activity of irisin on mitochondrial homeostasis (Fan et al., 2020). Irisin also activates the AMPK/UCP2 signaling pathway, which exerts a protective role on ischemia/reperfusion-induced renal injury (Zhang et al., 2020). Together, these studies imply that irisin may play an important role by influencing AMPK expression during ischemic stroke. However, considering that the literature related to the neuroprotective effect of irisin and mitochondrial dynamics in stroke is limited, further research is required to confirm this role.

## Discussion

Although treatment strategies for stroke have been explored over several decades, intravenous thrombolysis remains the primary and most effective method (Keizman et al., 2011). However, due to the limitation presented by the short window for treating stroke, many patients are not treated in time (Diop-Frimpong et al., 2011). According to a recent clinical study, the word “neuroprotection” should be replaced with “brain cytoprotection” because stroke affects the entire neurovascular unit and the underlying white matter (Savitz et al., 2019). Therefore, developing brain cytoprotectants in the context of thrombolysis along with pre-hospital/in-hospital/post-thrombolysis tactics is necessary. Alternative treatment strategies such as in-hospital pre-thrombectomy cytoprotection, as well as drugs targeting the ischemic cascade within neurons and the entire neurovascular unit to limit and prevent the expansion of the ischemic core still need to be explored (Savitz et al., 2019).

Irisin is reportedly induced by physical exercise to augment energy expenditure, according to the initial report (Boström et al., 2012). A large number of clinical and experimental investigations have subsequently confirmed that acute exercise induces the release of irisin into the blood. It should be noted that although there is still some conflicting evidence (Raschke et al., 2013; Albrecht et al., 2015), it is widely believed that irisin plays substantial roles in the pathophysiology of metabolic diseases. Moreover, irisin is not only a myokine but also an adipokine (Roca-Rivada et al., 2013). Thus, irisin may be a promising therapeutic bioactive hormone for the treatment of metabolic diseases. Recent studies have uncovered some important biological functions of irisin in other systems. For example, irisin regulates depression-like behavior (Wang and Pan, 2016). Due to the crosstalk between metabolic dysfunction and cardio-cerebrovascular diseases, the role of irisin in the cardio-cerebrovascular system is also a deeply studied research direction.

Irisin protects against endothelial injury and ameliorates atherosclerosis in Apo-E knockout mice (Lu et al., 2015). In the field of myocardial ischemia-reperfusion, the protective role of irisin via the regulation of the SOD2 and AMPK pathways has been demonstrated (Wang et al., 2018; Xin et al., 2020). Notably, AMPK, as the main transcription factor, is very important for the crosstalk between metabolic and cardio-cerebrovascular diseases. Consequently, irisin may exert a brain cytoprotective role by influencing the expression of AMPK.

Many scholars have also suggested that irisin is an exercise-induced muscle factor, with exercise promoting its large-scale expression in skeletal muscles, the heart, and the brain. These brain regions include Purkinje cells in the cerebellum (Varela-Rodríguez et al., 2016), astrocytes in the hippocampus (Piya et al., 2014), neurons in the brain (Wang et al., 2018), the hypothalamus (Dun et al., 2013), and even the CSF (Aydin et al., 2013). Irisin is therefore suggested to have a positive impact on the nervous system. In 2017, Li et al. first provided evidence that irisin is a neuroprotective hormone in cerebral ischemia, with its expression underlying the neuroprotective effects of physical exercise against cerebral ischemia. This finding provided strong evidence that irisin may exert a brain cytoprotective role during ischemic stroke.

Evidence suggests that irisin levels are affected by a large number of stressors. It is well-established that acute exercise increases the levels of blood irisin (Jedrychowski et al., 2015; Löffler et al., 2015; Samy et al., 2015). Two independent studies have demonstrated that serum irisin levels decrease between 1 and 24 h after heart ischemia in a mouse model (Bashar et al., 2018; Zhao et al., 2019). Another study has found that plasma irisin also decreases after ischemic stroke (Li et al., 2017), which suggests that the release of irisin from muscles into the blood is inhibited after ischemic stroke. Consistent with this perspective, levels of FNDC5, the precursor of irisin, are also decreased in skeletal muscles during cerebral ischemia. However, there is no literature exploring how ischemia affects FNDC5 expression and irisin secretion from skeletal muscles, and studies are just beginning to explore the potential mechanisms involved. Because of the limited literature about the role of irisin in ischemic stroke, further studies must be conducted in the future to elucidate the potential mechanism by which irisin confers its protective effect in stroke.

## Author Contributions

All authors listed have made a substantial, direct and intellectual contribution to the work, and approved it for publication.

## Conflict of Interest

The authors declare that the research was conducted in the absence of any commercial or financial relationships that could be construed as a potential conflict of interest.
